# Comparison of virtual reality and computed tomography in the preoperative planning of complex tibial plateau fractures

**DOI:** 10.1007/s00402-024-05348-9

**Published:** 2024-05-04

**Authors:** Christian Colcuc, Marco Miersbach, Miguel Cienfuegos, Niklas Grüneweller, Thomas Vordemvenne, Dirk Wähnert

**Affiliations:** 1 Bielefeld University, Medical School and University Medical Center OWL, Protestant Hospital of the Bethel Foundation, Department of Trauma and Orthopaedic Surgery, Burgsteig 13, 33617 Bielefeld, Germany; 2 Bielefeld University, Center for Cognitive Interaction Technology CITEC, Universitätsstraße 25, 33615 Bielefeld, Germany

**Keywords:** Virtual reality, Preoperative planning, Tibia plateau fracture

## Abstract

**Introduction:**

Preoperative planning is a critical step in the success of any complex surgery. The pur-pose of this study is to evaluate the advantage of VR glasses in surgical planning of complex tibial plateau fractures compared to CT planning.

**Materials and methods:**

Five orthopedic surgeons performed preoperative planning for 30 fractures using either conventional CT slices or VR visualization with a VR headset. Planning was performed in a randomized order with a 3-month interval between planning sessions. A standardized questionnaire assessed planned operative time, planning time, fracture classification and understanding, and surgeons’ subjective confidence in surgical planning.

**Results:**

The mean planned operative time of 156 (SD 47) minutes was significantly lower (*p* < 0.001) in the VR group than in the CT group (172 min; SD 44). The mean planning time in the VR group was 3.48 min (SD 2.4), 17% longer than in the CT group (2.98 min, SD 1.9; *p* = 0.027). Relevant parameters influencing planning time were surgeon experience (-0.61 min) and estimated complexity of fracture treatment (+ 0.65 min).

**Conclusion:**

The use of virtual reality for surgical planning of complex tibial plateau fractures resulted in significantly shorter planned operative time, while planning time was longer compared to CT planning. After VR planning, more surgeons felt (very) well prepared for surgery.

## Introduction

Tibial plateau fractures (TPFs) are rare, accounting for only 1% of all fractures. Complex proximal tibial plateau fractures are at risk for post-traumatic osteoarthritis and other long-term complications [1, 2]. Therefore, the primary goal is to restore optimal joint function by restoring articular surface congruency, overall joint stability, and proper load distribution [3]. However, the high variability of fracture patterns poses a challenge to surgical management. Therefore, accurate preoperative planning and fracture analysis are essential steps in the management of complex tibial plateau fractures. The first step in preoperative planning is the evaluation and classification of the tibial plateau fracture [4–6]. Computed tomography (CT) of the injured knee has led to increasingly accurate and detailed fracture classification. Thus, multiplanar visualization and assessment has become the standard in radiologic diagnosis and preoperative planning of these fractures [7]. Especially in recent years, advanced preoperative visualization of the fracture for surgical treatment planning has become a research focus. While 3D reconstruction of CT data is already explicitly recommended for treatment planning of tibial plateau fractures [8, 9], the current scientific focus is on 3D printing and virtual reality technologies [10, 11]. The use of 3D printing in preoperative planning for complex tibial plateau fractures provides better and more realistic information about the fracture, contributing to more precise planning based on accurate anatomical models. It also enables the creation of intraoperative navigation tools, such as drill guide sleeves for optimal screw positioning [12, 13]. Therefore, the implementation of point-of-care 3D printing may add value to the use of conventional imaging modalities alone. In particular, Dust et al. showed that the 3D printing group had higher interobserver reliability than CT and 3D CT alone in clinically less experienced readers [14]. However, it does requires time management and planning to implement this technology into daily clinical practice.

A further evolution of three-dimensional visualization is extended reality, which includes virtual, augmented, and mixed reality [11]. Virtual reality (VR) is entirely computer generated and aims to immerse the user in a “real” 3D environment. Therefore, VR glasses completely isolate the user from the outside world. Mixed Reality (MR) and Augmented Reality (AR) glasses allow interaction with the physical world. The main difference between MR and AR glasses is that MR provides the ability to interact directly with the holograms created, while AR limits the experience to visualization [15, 16]. 

The technology has evolved to become mature enough to be used commercially, including in medical applications. The case for integrating augmented reality technologies into the practice of orthopaedics and traumatology is convincing, especially given the expanding range of technically challenging procedures in the specialty [17, 18]. Despite the recognized potential, the adoption of Extended Reality technologies in orthopaedics and traumatology has unfortunately been behind other surgical specialties [19–21] and in particular the development of Extended Reality technologies for fracture surgery [11]. However, early clinical and preclinical studies suggest that Extended Reality technology has many advantages for many procedures in orthopedic and trauma surgery. Reduced operative time, reduced intraoperative x-ray exposure time, reduced intraoperative blood loss, and increased surgical precision are some of the key benefits demonstrated in these studies [22–26]. However, the first step in successfully treating a complex fracture is preoperative planning. To date, only one multicenter study has demonstrated an advantage of MRV over CT and 3D printing in preoperative planning and understanding of tibial plateau fractures [27]. Therefore, further application studies are needed to confirm the previous results and potentially provide further benefits in planning the treatment of complex tibial plateau fractures using extended rality technologies.

The purpose of this single-center study is to compare preoperative planning of complex tibial plateau fractures using virtual reality visualization versus computed tomography visualization. Specifically, planned operative time, planning time, fracture classification and understanding, and a survey of surgeon subjective confidence in surgical planning were measured.

## Materials and methods

### Study population

For this study, the Clinical Information System of our hospital was queried for the years 2015–2020. All patients with ICD 10 code S82.1X (fractures of the proximal tibia) were identified and exported to an Excel file. This research resulted in 158 potential cases. All of these cases were then reviewed by an experienced orthopaedic surgeon and a case collection was created. The following criteria were used for case selection: complex fracture morphology (AO 41 Type B and C), surgical treatment, appropriate imaging available (CT dataset). After selection using the above criteria, 40 eligible patients were identified for further processing. CT datasets were pseudonymized and exported from the radiology system for further workup.

### 3D model preparation

**Fig. 1:**
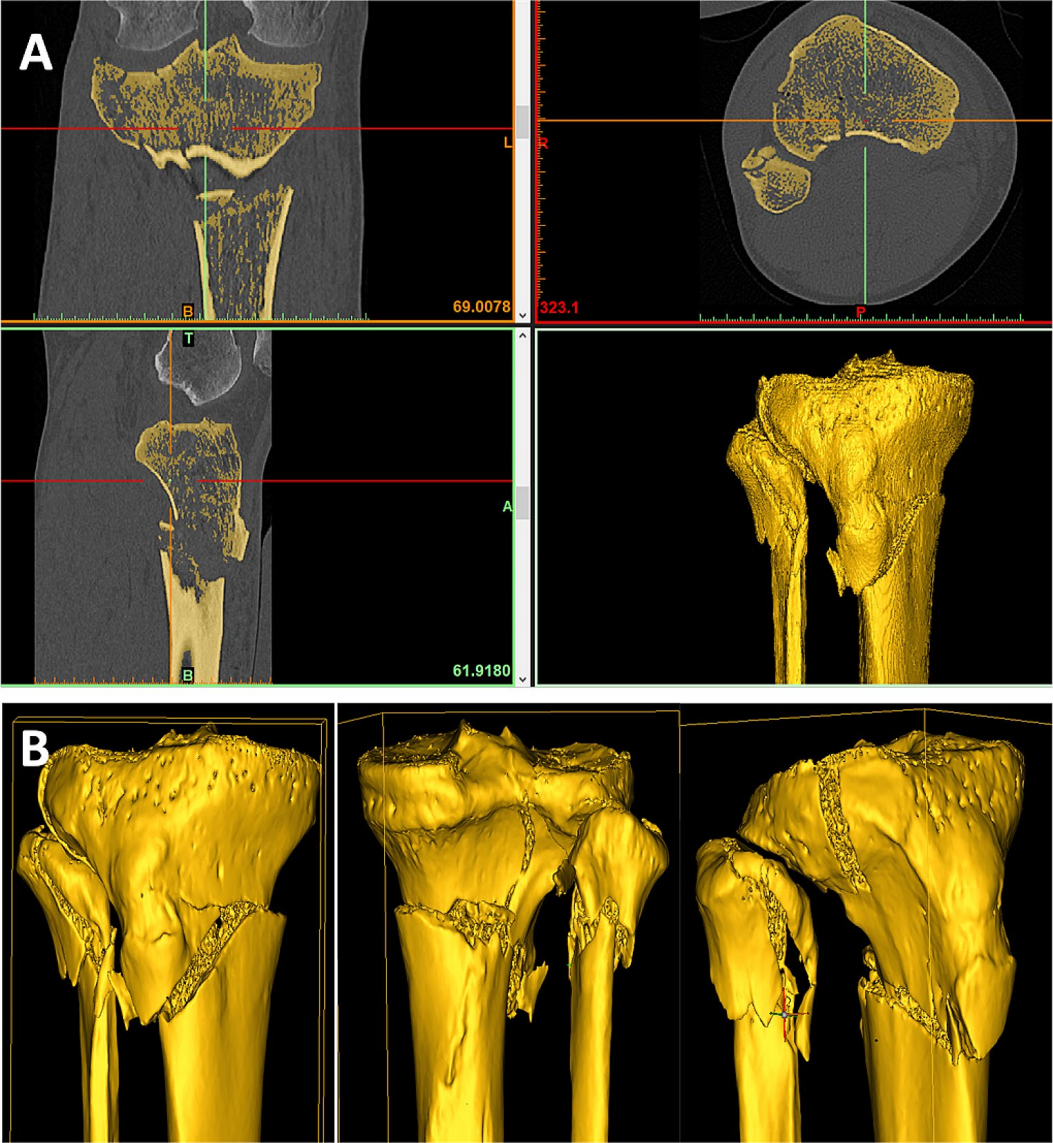
(**A**) Example of segmentation of a 3D model of a complex proximal tibia fracture. The yellow area represents the bone selected by the boundaries. (**B**) Final 3D model in 3 views

After exporting the CT data sets in DICOM (Digital Imaging and Communications in Medicine) format, they were imported into the OsiriX software (Pixmeo SARL, Bernex, Switzerland). The axial slices of the preoperative CT scan were used for further processing. A semi-automatic segmentation algorithm of the OsiriX software was used to segment the tibia. Thresholds were preset in the software to extract bony tissue. Due to intra- and inter-individual differences in the bone structure of the proximal tibia, manual control and adjustment of the upper and especially the lower Hounsfield unit thresholds was performed to obtain a meaningful model (Fig. 1). From the segmented bone, a 3D surface model of the proximal tibia of variable length (according to the fracture morphology) was generated using the OsiriX software algorithm. Finally, the 3D models were saved in STL (Standard Transformation Language) format for further use.

### Simulator platform

A state-of-the-art simulator platform has been developed to provide an immersive four-sided virtual environment for enhanced bone visualization. Custom-built using the Unity game engine, this platform is tailored to process and render high-resolution CT scans, presenting bone structures as free-floating 3D objects within the immersive environment. Designed specifically for the Oculus Quest 2 (Meta Platforms, Inc, California, USA), this wireless, self-contained system features a refresh rate of up to 90 Hz and delivers smooth and realistic visuals without the need for external sensors. This design ensures a seamless and uninterrupted user experience. In addition, navigation and interaction within this virtual world is facilitated by the Oculus Touch controllers, which allow for easy rotation of bone structures in three dimensions and the ability to zoom in for detailed inspection.

### Preoperative planning

After all 40 cases were prepared for preoperative planning, an uninvolved orthopedic surgeon reviewed all fractures in VR and CT visualization. Due to discrepancies or poor visualization quality of the fracture in VR, 10 cases were excluded from the final planning study, leaving 30 cases for the study.

Five experienced orthopaedic trauma surgeons of our department performed preoperative planning with both conventional (CT slices) and VR visualization, randomizing which modality was started. The order of the case was also randomized with a 3-month interval between planning sessions for each surgeon. A virtual reality headset (Meta Quest 2, Meta, Dublin, Ireland) was used for VR planning. CT-based planning was performed on a computer using the OsiriX DICOM viewer (Pixmeo SARL, Bernex, Switzerland), and axial, sagittal, and coronal slices could be used.

Surgeons were classified according to their experience in treating complex proximal tibial fractures in two groups experienced (up to 10 cases/year) and very erperienced ( more than 10 cases/year). A standardized surgical planning questionnaire, previously discussed with the surgeons, was completed during planning. To ensure standardized documentation and to avoid missing values, the questionnaire was completed by an orthopedic resident. The questionair included the following factors: AO classification and planned duration of surgery. In addition, the time required for planning was recorded. After each planning session, the surgeons were asked to rate on a scale of 1 to 6 the complexity of the fracture treatment, their satisfaction with the surgical preparation, the likelihood of being able to carry out the plan without changes, and the likelihood of full recovery of function.

### Data collection and statistical analysis

Once the data were digitized into an Excel spreadsheet (Microsoft 365, Redmond, USA), they were categorized (where necessary) for further analysis. Statistical analysis was performed using SPSS 25 (IBM Germany GmbH, Ehningen, Germany). A multiple regression analysis was performed for the variables planned operation time and planning time, and a logistic regression for the variable AO classification. This analysis was performed for the entire cohort and separately for the VR and CT planning groups. Krippendorff’s alpha coefficient was used to test for interrater and test reliability.

## Results

### Planned operating time

**Fig. 2:**
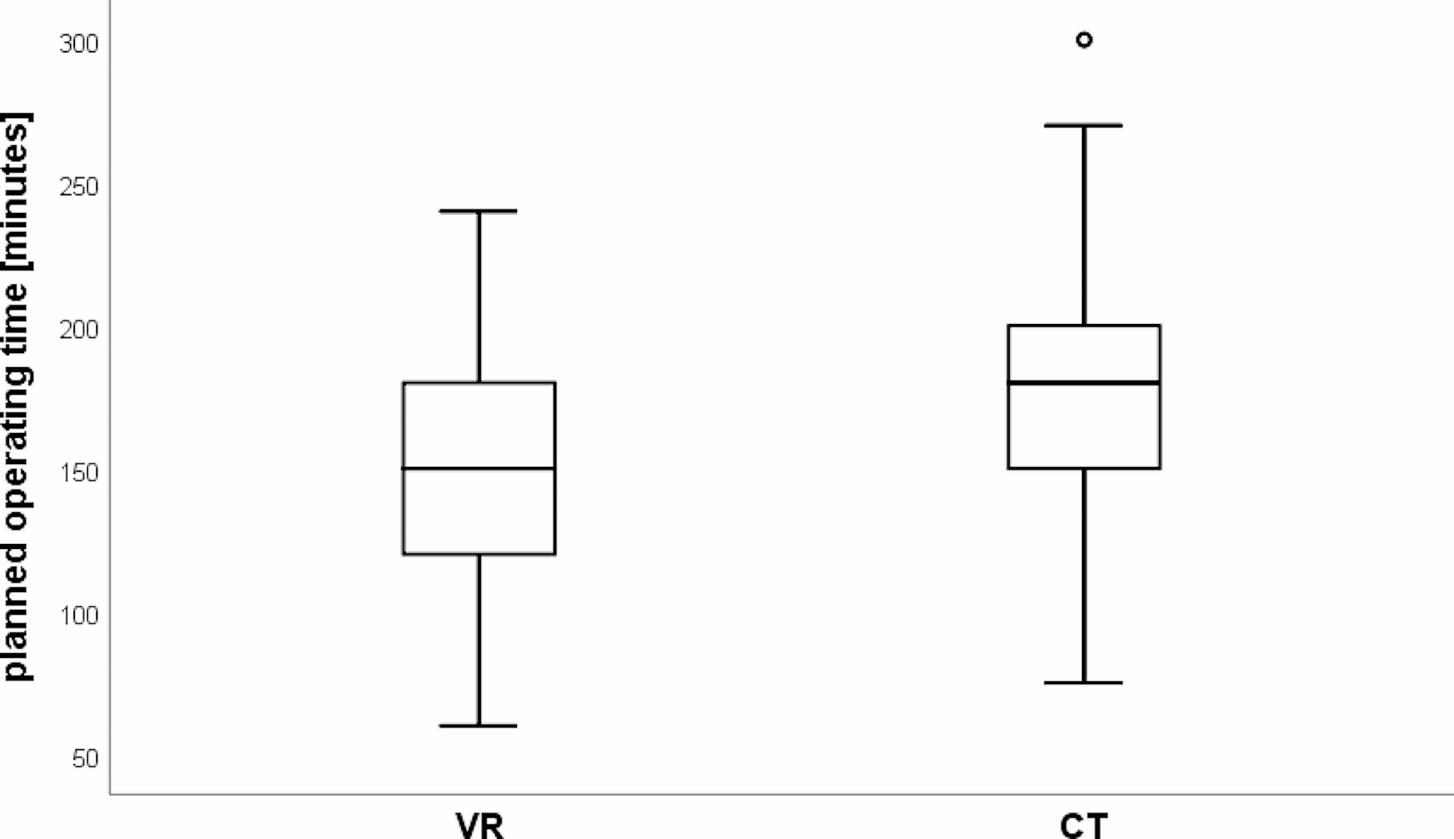
Box plots of planned operating time in the VR and CT groups

The mean planned operating time of 156 (SD 47) minutes in the VR group is significantly lower (*p* < 0.001) than in the CT group (172 min; SD 44). The mean planned operating time in the VR group is 9.5% less than in the CT group (Fig. 2).

In addition to the type of planning (VR vs. CT), the experience of the planning surgeon in the treatment of complex tibial plateau fractures is a significant parameter influencing the planned operative time in the overall collective. As the level of experience increases, the planned operative time decreases by approximately 11 min. The severity of the fracture according to the AO classification (B vs. C) also has a significant influence on the planned operating time, with an average of 18 min more planned operating time for C fractures. In the VR group, only the experience of the surgeon, the complexity of the fracture (AO type) and the complexity of the treatment show a significant influence on the planned operative time. Results are shown in Table 1.

**Table 1:** Summary of the multiple regression analysis of planned operating time [minutes] for all cases, for the VR group and for the CT group (significance < 0.05 marked in bold)

	All cases				VR-group				CT-group			
		Sig.	95.0% confidence intervals for B			Sig.	95.0% confidence intervals for B			Sig.	95.0% confidence intervals for B	
	Regression coefficient B		Lower limit	Upper limit	Regression coefficient B		Lower limit	Upper limit	Regression coefficient B		Lower limit	Upper limit
Planning (VR vs. CT)	10,62	**< 0,001**	4,88	16,35								
Experience of surgeon	-11,58	**< 0,001**	-15,22	-7,94	-21,61	**< 0,001**	-26,15	-17,06	-2,04	0,46	-7,46	3,39
AO classification (B vs. C)	17,73	**< 0,001**	11,23	24,23	16,27	**< 0,001**	8,65	23,90	13,66	**0,01**	3,52	23,80
Fracture complexity	14,75	**< 0,001**	9,75	19,75	16,50	**< 0,001**	10,33	22,66	15,30	**< 0,001**	8,03	22,58
Quality of the preparation	-6,85	**< 0,001**	-11,47	-2,23	-5,18	0,11	-11,54	1,18	-10,25	**< 0,001**	-16,52	-3,99
Implement plan without change	3,16	0,20	-1,62	7,93	4,01	0,23	-2,53	10,56	0,22	0,94	-6,05	6,49
Restore function	-5,16	**0,01**	-9,21	-1,12	-1,65	0,55	-7,14	3,84	-3,43	0,22	-8,95	2,10

### Planning time

The time required for preoperative planning differed significantly between groups (*p* = 0.027). The mean planning time in the VR group was 3.48 min (SD 2.4), 17% longer than in the CT group (2.98 min, SD 1.9).

In addition to the planning modality (VR vs. CT), the most relevant parameters influencing the planning time are the surgeon’s experience (-0.61 min) and the complexity of the fracture treatment (+ 0.65 min).

Looking at the VR group, surgeon experience is not a significant parameter for planning time, whereas fracture type according to AO classification significantly influences planning time in this group (C-fractures + 0.92 min) (Table 2).

**Table 2:** Summary of the multiple regression analysis of the planning time [minutes] for all cases, for the VR group and for the CT group (significance < 0.05 marked in bold)

	All cases				VR-group				CT-group			
		Sig.	95.0% confidence intervals for B			Sig.	95.0% confidence intervals for B			Sig.	95.0% confidence intervals for B	
	Regression coefficient B		Lower limit	Upper limit	Regression coefficient B		Lower limit	Upper limit	Regression coefficient B		Lower limit	Upper limit
Planning (VR vs. CT)	-0,45	**0,03**	-0,84	-0,05								
Experience of surgeon	-0,61	**< 0,001**	-0,87	-0,35	-0,43	0,06	-0,88	0,02	-0,69	**< 0,001**	-1,02	-0,36
AO classification (B vs. C)	0,32	0,17	-0,14	0,78	0,92	**< 0,001**	0,30	1,55	-0,16	0,63	-0,79	0,48
Fracture complexity	0,65	**< 0,001**	0,30	1,00	0,16	0,55	-0,36	0,68	1,11	**< 0,001**	0,63	1,58
Quality of the preparation	0,12	0,44	-0,19	0,44	0,16	0,53	-0,34	0,65	-0,25	0,21	-0,65	0,14
Implement plan without change	0,11	0,50	-0,21	0,43	0,02	0,93	-0,48	0,53	0,09	0,66	-0,30	0,47
Restore function	-0,12	0,41	-0,39	0,16	-0,65	**< 0,001**	-1,07	-0,22	0,37	**0,04**	0,03	0,70
Planned operating time	0,00	0,88	-0,01	0,01	0,00	0,89	-0,01	0,01	-0,01	0,23	-0,02	0,00

### AO classification

The different planning modalities also resulted in a change in fracture type classification. The mean classification type differs in 8 cases when comparing VR and CT planning. Interestingly, a downgrading from type C to type B fractures was observed in VR planning in 7 cases. Only 1 fracture was upgraded from type B to type C in VR planning.

In terms of interrater reliability, a Krippendorff’s alpha of 0.801 for virtual reality planning showed very good agreement among the 5 raters for fracture classification. Using CT planning, the Krippendorff’s α was only 0.605. When comparing the two planning methods, the Krippendorffs α was 0.67, indicating moderate agreement.

### Quality of preoperative planning

**Fig. 3:**
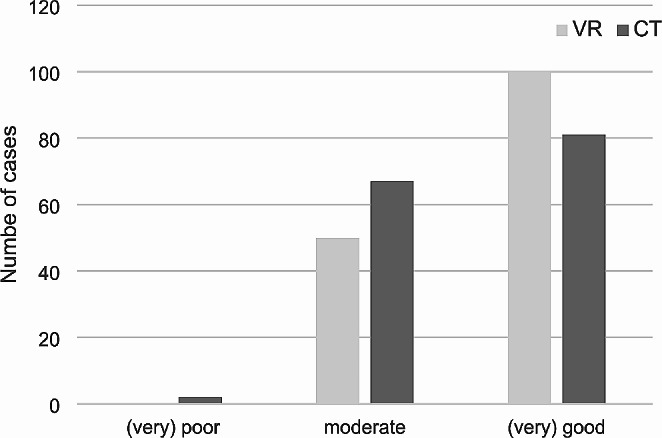
The graph shows surgeon satisfaction with preoperative planning in the VR and CT groups

At the end of each planning session, surgeons were asked to rate how well they felt prepared for the surgery (Fig. 3). The results show that no surgeon in the VR group felt (very) poorly prepared. Overall, 67% of the VR group and 54% of the CT group felt (very) well prepared.

## Discussion

The main finding of this study was that the planned operative time was significantly shorter in the VR group than in the CT group. The operating time is an essential parameter which, as it increases, also means an increase in the risk of complications such as infections. Colman et al. showed that operative times longer than 3 h and open fractures are associated with an increased overall risk of surgical site infection after open tibial plateau plating [28]. Therefore, operative time is an important prognostic factor for patient safety and outcome. Our results show that the planned operating time can serve as a valid parameter. Our results show that the planned operating time can serve as a valid parameter, as it is on average 11.5 min shorter for experienced surgeons and on average 17 min longer for type C fractures. Interestingly, our results show that particularly experienced surgeons benefit from the VR technique, planning on average 21.6 min less operating time with VR technology than less experienced colleagues. In contrast, the difference in CT planning is only 2 min. This shows that experienced surgeons in particular can use the additional information provided by VR technology for surgical planning. On the other hand, fracture classification and the complexity of the fracture have similar effects on the planned operating time in both groups. Although this study did not evaluate the translation of these findings to actual operative time, these theoretical results in VR planning highlight the individual surgeon’s better understanding of fracture morphology.

Another important factor is the planning time required. Time is a valuable commodity in the medical profession, so new techniques are also assessed in terms of their time requirements. This can be decisive for the success or failure of an innovation. None of the surgeons in this study had any previous experience with VR, so it is not surprising that surgical planning with the new technique took longer. The difference of 30 s on average can be overcome through training and regular use. A newly established technique always requires a learning curve, which varies from surgeon to surgeon and can have a significant impact on planning time. We therefore agree with Bitschi et al. that the regular use of VR technology in surgical planning will bring further benefits in terms of fracture understanding and planning time [27].

Overall, the use of VR technology for planning complex tibial plateau fractures showed advantages over planning with CT images. Our results are consistant with the only study to our knowledge that also examined the use of extended reality for preoperative planning of complex tibial plateau fractures [27]. Bitschi et al. investigated whether mixed reality visualization (MRV) using mixed reality glasses could provide an advantage over CT and/or 3D printing in preoperative planning for complex tibial plateau fractures [27]. They used 3 complex tibial plateau fractures for their study and presented them to 23 experienced surgeons as a CT dataset, a 3D printed model, or with augmented reality technology. A standardized questionnaire on fracture morphology and treatment strategy was completed after each imaging session. They could show that preoperative mixed reality visualization of complex tibial plateau fractures leads to increased certainty in fracture understanding and the planned treatment strategy, as well as a higher detection rate of fractures in the posterior segments.

In addition, the authors showed that 82.1% of participants rated MRV as advantageous compared to CT for fracture morphology and and treatment planning. However, this study also had limitations. The small number of fractures and the lack of randomization of fracture presentation and the presentation of the fracture three times leads to a more intensive study of the fracture and a certain bias  [27]. To somehow overcome this limitation, we conducted our study using 30 different tibial plateau fractures with 5 surgeons from the same level I trauma center. Case planning was performed in random order with a 3-month interval between planning sessions to avoid habituation and more intensive fracture processing.

Another important factor in fracture understanding and surgical planning is the classification of tibial plateau fractures. Several studies have shown that the intra- and interrater reliability of classification systems for tibial plateau fractures is higher with CT imaging than with conventional radiography [7]. Further improvement in the interobserver reliability of any classification system has been achieved with the introduction of three-dimensional CT [27, 29, 30]. Extended reality visualization can be considered as a digital evolution of 3D CT. The design used in this study provides a seamless and uninterrupted user experience. In addition, navigation and interaction in this virtual world is facilitated by the Oculus Touch controller. The virtual bone model can be freely rotated and turned in all directions. Regarding the classification of fractures, it was shown that the evaluation of CT images led to an overestimation of fractures in our collective, whereas the evaluation using VR glasses led to a lower classification of fractures within the AO classification in 7 cases. Bitschi et al. used different classification systems of tibial plateau fractures in their study. In 7%, the Schatzker classification changed after mixed reality visualization compared to CT visualization. In the 10-segment classification, mixed reality visualization caused a change in the selected segments in 79%. Thus, visualization using augmented reality techniques leads to increased confidence in fracture understanding [27]. 

Overall, 67% of the surgeons in the VR group felt (very) well prepared for surgery due to the visualization provided. None of the surgeons felt poorly prepared. A possible reason for this could be a better understanding of the fracture due to the possibility of improved fracture visualization. This is consistent with the study by Bitschi et al., who found a benefit of mixed reality visualization over CT in 82.1% of participants [27]. As an alternative, 3D printing could be used in daily practice. However, this is a costly and time-consuming process. Both the conversion of data from CT and some manual sequencing add up to several hours of time. The purchase and maintenance of the 3D printer as well as the printing material also make the procedure not cost-effective [27, 31, 32]. VR of the CT data on the VR goggles is performed by the software in seconds without any manual editing, so a direct comparison with a 3D print was not made in this study. Bitschi et al. who did this comparison found no change in Schatzker classification comparing mixed reality visualization and 3D printing. In the 10-segment classification, an additional change in Schatzker segments was found in 43% of the segments comparing mixed reality visualization and 3D printing. An additional benefit of 3D printing was seen by 57% of the participants. No significant difference in perceived confidence in understanding fracture morphology was found between mixed reality visualization and 3D printing [27]. 

While the simulator platform has made significant strides in bone visualization, there are several potential enhancements that could further refine the user experience in subsequent releases. Annotation tools could be introduced to allow surgeons to mark specific points, add notes, and sketch directly on the 3D model for more interactive and detailed analysis. Other tools, such as measurement tools, would provide surgeons with the ability to accurately quantify fracture gaps, angles, and other important dimensions. Simulation capabilities would also allow surgeons to visualize potential interventions, such as screw or plate placement, prior to the actual procedure. In addition, the integration of AI-driven suggestions based on fracture complexity could further refine preoperative strategizing [33].

Additionally, the integration of eye-tracking technology represents a promising avenue for future development. Eye-tracking could provide surgeons with a more intuitive way to navigate and interact with the virtual environment. As highlighted by Zheng et al., the spatiotemporal characteristics of surgeons’ eye-hand coordination can be used to assess the level of surgical experience [34]. Wu et al. found that eye-tracking metrics such as pupil diameter and gaze entropy were sensitive to changes in workload, suggesting the potential of eye-tracking to measure perceived workload during surgical tasks [35].

In summary, and in light of these findings, VR technology offers multiple advantages and opportunities in its use for surgical preparation and planning, and offers opportunities for further development in the future. The first step is to prospectively validate the findings in a randomized trial comparing the two planning modalities. It should be investigated to what extent the theoretically planned shorter surgical time with VR glasses can actually be confirmed intraoperatively.Furthermore, it could be investigated whether the complication and revision rate of fractures can be reduced by VR technology.

This study also has several limitations. First, there was no control of the variable “planned operative time” and no evaluation of the validity of the VR technique. Therefore, the findings of perceived good surgeon preparation and shorter estimated operative time cannot be extrapolated to real-world data. To somehow standardize the study, the surgeon’s task was to plan the operation under the best soft tissue conditions (no open fractures, no soft tissue damage), which is not the reality in many complex proximal tibial fractures. Therefore, the comparison with the real operating time will not provide a meaningful result and was not performed. In addition, we had to exclude 10 cases due to insufficient fracture visualization in virtual reality. This is due to the retrospective case selection, in some cases only low quality CT scans were available, so the 3D virtual reality model did not represent the fracture. Another relevant point is the evaluation of subjective values such as planned operative time. We chose this procedure first to gain experience and to evaluate the acceptance and performance of this system in a clinically relevant application. We believe that the planning of 30 different fractures by five surgeons with different levels of experience in treating tibial plateau fractures provides representative data.

## Conclusion

The use of virtual reality for surgical planning of complex tibial plateau fractures resulted in significantly shorter planned operative time, while planning time was longer compared to CT planning. After VR planning, more surgeons felt (very) well prepared for surgery. Therefore, the results suggest that the use of virtual reality vizualization for surgical planning of complex tibial plateau fractures appears to offer advantages over 2D CT analysis. However, due to the retrospective nature of the study, we were only able to evaluate subjective data. Nevertheless, we were able to show that this system works in a clinical application and is accepted by surgeons. Further research should focus on the transferability of the results in a prospective study.
